# Short-chain fatty acids propionate and butyrate control growth and differentiation linked to cellular metabolism

**DOI:** 10.21203/rs.3.rs-3935562/v1

**Published:** 2024-02-16

**Authors:** Michael Nshanian, Benjamin S. Geller, Joshua J. Gruber, Faye Chleilat, Jeannie Marie Camarillo, Neil L. Kelleher, Yingming Zhao, Michael P. Snyder

**Affiliations:** 1 Department of Genetics, Stanford University, School of Medicine, Stanford, CA;; 2 Department of Chemistry, Molecular Biosciences and Proteomics Center of Excellence, Northwestern University, Evanston, IL;; 3 Department of Biochemistry and Molecular Genetics, Feinberg School of Medicine, Northwestern University, Evanston, IL;; 4 Ben May Department of Cancer Research Committee on Cancer Biology, University of Chicago; Chicago, IL;; 5 Center for Genomics and Personalized Medicine, Stanford University School of Medicine, Stanford, CA

## Abstract

The short-chain fatty acids (SCFA) propionate and butyrate are produced in large amounts by microbial metabolism and have been identified as unique acyl lysine histone marks. In order to better understand the function of these modifications we used ChIP-seq to map the genome-wide location of four short-chain acyl histone marks H3K18pr/bu and H4K12pr/bu in treated and untreated colorectal cancer (CRC) and normal cells, as well as in mouse intestines *in vivo*. We correlate these marks with open chromatin regions along with gene expression to access the function of the target regions. Our data demonstrate that propionate and butyrate act as promoters of growth, differentiation as well as ion transport. We propose a mechanism involving direct modification of specific genomic regions, resulting in increased chromatin accessibility, and in case of butyrate, opposing effects on the proliferation of normal versus CRC cells.

Histone post-translational modifications (PTMs) may mediate crucial interplay between epigenetics and metabolism with important consequences for human health and disease. In addition to canonical lysine acetylation, eight types of short-chain lysine acylations have been recently identified on histones, such as propionylation (Kpr), butyrylation (Kbu), 2-hydroxyisobutyrylation (Khib), succinylation (Ksucc), crotonylation (Kcr) and β-hydroxybutyrylation (Kbhb)^1–3^. A growing body of evidence points to a unique epigenetic regulatory role for each of these modifications^4–6^. Their presence on histones is determined by the cellular metabolic state and the availability of various forms of acyl-CoA, linking metabolism to epigenetic regulation^4,7^. The cellular concentrations of non-acetyl acyl-CoAs in turn are dependent on the presence of SCFAs. Treating cells with heavy isotope-labeled SCFAs leads to heavy acyl labeling on histone proteins, pointing to a conversion of SCFAs to their cognate acyl-CoAs that are used as cofactors in histone acylation reactions^4,8–10^. This is accompanied by concomitant increases in the steady-state level of respective histone acylations in a dose-dependent manner^4,9,11^.

Recent studies show that major families of histone acetyl transferases (HATs) can catalyze histone acylation using acetyl-, propionyl- and butyryl-CoA cofactors with similar efficiencies^12–14^. For most HATs, the preference for the competing cofactor largely depends on the size of the acyl donor chain^13^. Furthermore, although almost all HATs strongly prefer acetyl-CoA, bulk levels of lysine acylations can be induced in a dose-dependent manner, in response to increasing levels of given acyl-CoA metabolites^14^. Moreover, cellular concentrations of various acyl-CoAs span orders of magnitude and are closely correlated with relative abundances of acyl marks identified *in vivo*^14^. There is also evidence that acyl-CoAs can acylate histones non-enzymatically *in vitro*^14^. These findings point to a direct link between cellular metabolism and epigenetic regulation where differential acylation is driven by cellular concentrations of respective metabolic substrates^14–16^.

Histone acetyl marks are generally associated with active regulatory elements (effects in *cis*) that promote gene expression by neutralizing Lys positive change, leading to electrostatic and structural changes in chromatin and the recruitment of readers of acylation (effects in *trans*). Chromatin immunoprecipitation followed by sequencing (ChIP-seq) experiments on Kbu, Khib, Kbhb, and Kcr show an association of active regulatory elements with histone acylations and their relative levels^2,8,9,11,17^. Recent evidence demonstrates that differential acylation states correlate with distinct physiological states and biological processes involving signal dependent gene activation, development and metabolic stress^2,8,9,11,18,19^. With respect to metabolic regulation of histone acylation, evidence points to a regulatory mechanism that favors non-acetyl acylation under low-glucose conditions^20^. Under the latter, the levels of acetyl-CoA will be reduced leading to ketogenesis, high NAD+/NADH ratio and activation of ACSS2, a known source of acyl-CoAs^11,21,22^. Acylation then occurs with acyl-CoAs other than acetyl-CoA.

Two of the SCFAs involved in histone acylation, propionate and butyrate, are generated in large quantities by microbial metabolism (as high as 70 – 100 mM in the gut lumen) and contribute to a wide array of cellular processes^23,24^. They can act as sources of energy, or as substrates for histone acylation and chromatin modification by directly targeting sites or recruiting remodeling proteins^5^. While the underlying regulatory mechanisms are largely unknown, the histone PTM state plays a key role^4,5^. Importantly, the abundance of SCFAs in the microbiome from dietary fiber metabolism makes them very attractive natural therapeutic agents, especially in the context of CRC. In cancer cells, butyrate and to a lesser extent propionate have been shown to have anti-proliferative properties that are generally attributed to inhibition of histone deacetylation (HDAC)^25,26^. According to this model, the antiproliferative, apoptotic properties of SCFAs in cancer cells are caused by histone hyperacetylation resulting from HDAC inhibition^27^.

In this study, we sought to determine the regulatory function of several short-chain acyl lysine histone marks, namely propionyl and butyryl H3K18 and H4K12 in CRC versus normal cells, and *in vivo*, as well as the effect of propionate and butyrate supplementation on chromatin accessibility and transcription (Fig. 1a). We also examined global acetylation levels as a function of propionate supplementation. We show that unique histone marks H3K18pr/bu and H4K12pr/bu are associated with genomic regions distinct from their acetyl counterparts. On a genome-wide level, they associate with targets controlling growth, differentiation and unfolded protein response. Furthermore, they result in a more open chromatin structure and lead to the recruitment of a broad range of transcription factors (TFs). In the context of CRC cells, high concentrations of SCFAs (≥ 10 mM), especially butyrate, ultimately lead to the overexpression of key oncogenes and dysregulation of homeostatic processes like cell cycle and DNA replication. By contrast, in normal cells and in fiber-supplemented mouse intestines, Kbu marks associate with cell-substrate junction assembly, ion transport, and cell proliferation. These results yield insights into how dietary factors, microbial metabolism and epigenetics are integrated to modulate tissue physiology and cancer susceptibility.

## RESULTS

### Identification of CRC H3K18pr/bu and H4K12pr/bu marks

We focused on H3K18 and H4K12, since acetylation at these sites has been associated with poor outcome in CRC^28–30^. To test for propionylation and butyrylation at these sites we treated CRC cells with increasing levels (0 – 10 mM) of sodium propionate (NaPr) and sodium butyrate (NaBu), consistent with physiological levels of these SCFAs in fiber-supplemented colon^31,32^ (range 1 – 100 mM). We then probed for the presence of these marks in acid-extracted histones using modification-specific antibodies. Immunoblots indicate the presence of propionylation and butyrylation on H3K18 and H4K12 at 10 mM, and in case of H3K18pr, at 1 and 10 mM NaPr treatment (Supplementary Fig. 1a, b). Dot blot assays against synthetic peptides containing acyl chains of varying length showed high specificity. The results confirmed the conversion of NaPr and NaBu to their cognate acyl-CoAs and their deposition as propionyl and butyryl marks on H3K18 and H4K12 in a dose-dependent manner. To further test for the presence of lysine propionylation on histones H3 and H4, we treated CRC cells with ^13^C-labeled NaPr and searched for heavy peptides containing modified sites of interest by tandem mass spectrometry (MS/MS).

Our results clearly indicate a direct relationship between NaPr supplementation and lysine propionylation on H3 and H4 (Supplementary Fig. 2). At 10 mM supplementation, the amount of propionylation on H3 K18 and K23 increased 1.84-fold (*P* < 0.01) and 2.86-fold (*P* < 0.05), respectively, compared to the control (Supplementary Fig. 2a, b). With respect to H4, our results showed a dose-dependent rise in propionylation at K12, K16, K8, and to a lesser extent K5 at 10 mM supplementation (*P* < 0.05) (Supplementary Fig. 2c). These data indicate the uptake of NaPr and its deposition onto histones H3 and H4 as propionyl lysine marks. We then examined acetylated versus unmodified states on H3 and H4 to compare the relationship between the two PTM states after NaPr treatment. Our results do not show a significant change in the levels of acetylation on H3K18 or H4K12 (data not shown). The most abundant states on H3 and H4 were the unmodified ones, with a ratio of the unmodified to acetylated states approximately 10:1. This indicates that NaPr supplementation does not directly lead to increased histone acetylation at these sites.

To test whether SCFA supplementation resembled HDAC inhibition we compared treatment of CRC cells with increasing levels of NaPr, NaBu, and trichostatin A (TSA), a potent HDAC inhibitor. TSA treatment led to loss of cell viability at 0.1 μM and near complete cell death at 1 μM treatment (*P* < 0.01) (Supplementary Fig. 3). Butyrate, with its much weaker HDAC activity compared to TSA, resulted in reduced cell viability only at 10 mM supplementation (*P* < 0.0001). Propionate on the other hand, did not affect cell viability in the 0 – 10 mM range but only at 100 mM (*P* < 0.0001). Both NaPr and NaBu supplementation resulted in near complete loss of cell viability at 100 mM concentration. Because of the toxicity apparent at high levels, our experiments were performed with 10 mM NaPr and 1 mM NaBu to avoid cytotoxicity artifacts.

### Genomic localization of H3K18pr and H4K12pr in CRC cells reveals targets involved epidermal growth, transport and unfolded protein response

**Fig. 1 |:**
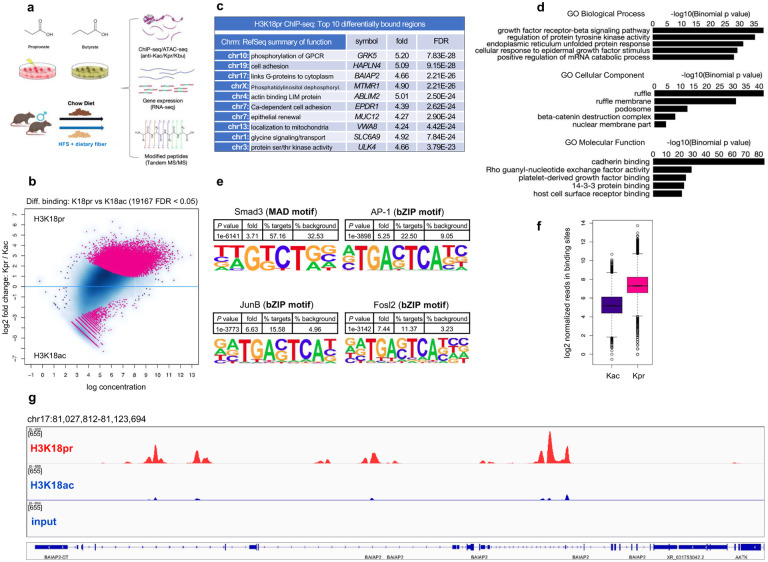
Genome-wide H3K18pr distribution. **a** SCFAs as regulatory elements: experimental design overview. **b** H3K18pr vs H3K18ac differential binding at 10 mM NaPr treatment. Sites identified as significantly differentially bound are shown in red. n = 2 experimental replicates for each mark and input. **c** Top ten differentially bound regions associated with H3K18pr, annotated to within 1 Kb of TSS, sorted by false-discovery rate adjusted *P* value (FDR < 0.05). **d** Top GO Biological Process, Cellular Component and Molecular Function terms of H3K18pr-associated *cis*-regulatory elements (5+ 1 Kb) determined by GREAT. **e** Differential motif analysis of H3K18pr vs H3K18ac peaks analyzed using HOMER. *P* values were determined by binomial test. **f** Normalized reads in H3K18pr vs H3K18ac-associated binding sites at 10 mM NaPr treatment. **g** Signal tracks for regions representing *BAIAP2*. Signal intensity of peaks in 95 Kb-spanning *BAIAP2* region showing H3K18pr vs H3K18ac binding at 10 mM NaPr treatment with input as background.

We next focused on the genome-wide distribution of H3K18pr and H4K12pr. Given the close coupling of acetylation, propionylation and butyrylation, we have compared differential Kpr binding to the corresponding acetyl marks. Results showed that out of 19,167 sites identified as differentially bound after NaPr treatment, 17,299 (90%) were associated with H3K18pr versus 1,868 associated with H3K18ac (FDR < 0.05) (Fig. 1b, c, g). Gene Ontology (GO) analysis of Kpr-bound regions and distal *cis*-regulatory elements^33^ pointed to enrichment in epidermal growth factor stimulus and cadherin binding, as well as endoplasmic reticulum (ER) unfolded protein response (Fig. 1d). KEGG pathway analysis also showed enrichment focal adhesion (110), actin cytoskeleton (115) and cancer regulatory pathways (Supplementary Fig. 4a, b). We focused on enrichment of CRC-relevant motifs such as SMAD2/3 of TGF-β pathway, as well as AP-1, FOSL2 and JUNB. All four motifs showed enrichment in K18pr over K18ac and input, with JUNB, FOS2L and AP-1 exhibiting greater than a six, seven and five-fold enrichment over background, respectively (Fig. 1e). Differential binding of key Wnt/β-catenin pathway genes showed ~3-fold enrichment in *CTNNB1*, *TCF20*, *LEF1* (Supplementary Fig. 4c). We also observed a 2–3-fold enrichment in *FOS* and *JUN*. Distribution of reads over all differentially bound sites showed Kpr as having a higher mean read concentration compared to Kac (Fig. 1f). Hierarchical clustering of Kpr annotated genes showed several clusters including cell projection organization and localization, while chromosomal positions and distribution by gene type showed enriched regions on chromosomes 3, 9, 17, 19, as well as the presence of lncRNAs and processed pseudogenes (Supplementary Fig. 4d-f).

**Fig. 2 |:**
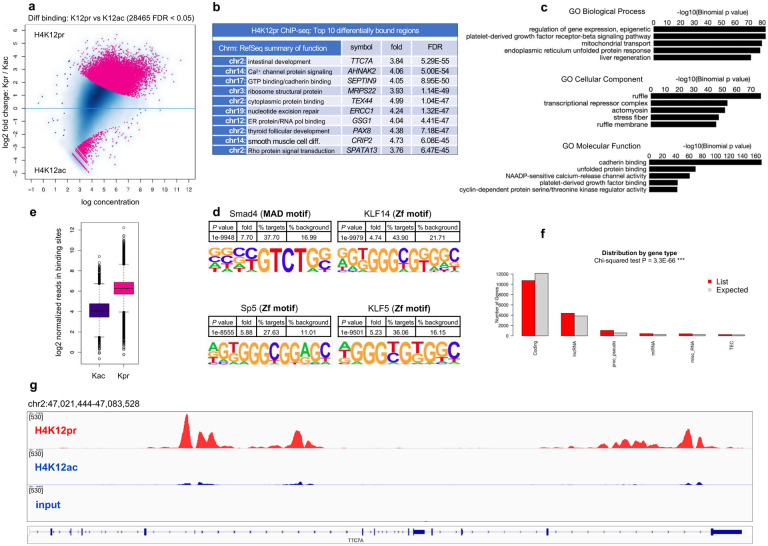
Genome-wide H4K12pr distribution. **a** H4K12pr vs H4K12ac differential binding at 10 mM NaPr treatment. Sites identified as significantly differentially bound are shown in red. n = 2 experimental replicates for each mark and input. **b** Top ten differentially bound regions associated with H4K12pr, annotated to within 1 Kb of TSS, sorted by false-discovery rate adjusted *P* value (FDR < 0.05). **c** Top GO Biological Process, Cellular Component and Molecular Function terms of H4K12pr-associated *cis*-regulatory elements (5+ 1 Kb) determined by GREAT. **d** Differential motif analysis of H4K12pr vs H4K12ac peaks analyzed using HOMER. *P* values were determined by binomial test. **e** Normalized reads in H4K12pr vs H4K12ac-associated binding sites at 10 mM NaPr treatment. **f** Distribution of H4K12pr peaks by gene type with *P* value measured by Chi-squared test. **g** Signal tracks for regions representing *TTC7A*. Signal intensity of peaks in 95 Kb-spanning *TTC7A* region showing H4K12pr vs H4K12ac binding at 10 mM NaPr treatment with input as background.

H4K12pr ChIP-seq identified 28,465 sites as differentially bound between Kpr and Kac, with 27,175 (95%) sites associated with Kpr (FDR < 0.05) (Fig. 2a, b, g). Genomic regions annotation pointed to enrichment in genes controlling Rho-guanyl-nucleotide exchange activity and differentiation, cadherin and ER protein binding, as well as calcium channel signaling (Fig. 2b). As with H3K18pr, GO and KEGG analysis pointed to pathways controlling platelet-derived growth factor and cadherin binding, ER unfolded protein response, and Ca^2+^ channel activity (Fig. 2c, Supplementary Fig. 5a, b). Motif analysis also showed enrichment in the SMAD family and Krüppel-like factors KLF5, KLF14 known for their regulatory role as recruiters of other TFs (Fig. 2d). Hierarchical clustering showed several clusters such as cellular catabolic processes, protein transport and cellular localization, while chromosomal positions and distribution by gene type showed enrichment along chromosomes 7, 9, 11, 17, as well as the presence of long non-coding RNAs (lncRNAs) and processed pseudogenes (Supplementary Fig. 5d, e, Fig. 2f). H4K12pr peak distributions, regulated genes, GO enrichments analysis showed generally similar results with H3K18pr analyses, including prioritization of Wnt/β-catenin, TGF-β and FOS, JUN, MYC TFs (Figs. 2a, c, e, Supplementary Fig. 5c).

We then integrated our Kpr ChIP-seq results with assay for transposase-accessible chromatin followed by sequencing (ATAC-seq) and RNA-seq results in CRC cells to determine intersecting genomic coordinates and overlapping annotated genes. Genomic coordinates present in both Kpr ChIP-seq and propionyl ATAC-seq data sets (n = 4391*, P* = 4.75e-4 and n = 4038, *P* = 2.31e-38, respectively) showed enrichment in β-catenin-TCF complex assembly, negative regulation of MAPK cascade, and actin filament organization (Supplementary Fig. 6a, b, e, f). There was also enrichment in Rho guanyl-nucleotide exchange factor activity and proteins involved in cell adhesion. Genes relevant to the identified pathways, and CRC in particular, also showed increased accessibility by ATAC-seq, indicating more open chromatin structure following NaPr treatment (Supplementary Fig. 6c, d, g, h).

Integration with propionyl RNA-seq showed overlap between both H3K18pr and H4K12pr targets and upregulation (n = 1528, *P* = 7.10e-154 and n = 1585, *P* = 1.69e-147), as well as downregulation (n = 1015, *P* = 3.29e-12 and n = 1317, *P* = 1.35e-167) of gene expression, respectively (Supplementary Fig. 7a, c, e, g). GO analysis of Kpr targets and upregulated genes identified pathways in differentiation, localization as well as actin filament-based processing, while downregulated genes were involved in mitotic cell cycle, RNA metabolism and processing (Supplementary Fig. 7b, d, f, h). This points to a mechanism through which propionate affects the CRC epigenetic regulatory landscape to promote growth, differentiation, and localization over regulation of cell cycle and RNA processing.

### Genomic localization of H3K18bu and H4K12bu in CRC cells reveals targets involved in epithelial growth and differentiation

We next examined Kbu marks on the same sites to gain functional insight and observe NaBu treatment (1 mM) effects on chromatin structure and accessibility. Butyrate has been shown to activate the TGF-β pathway^34^ and the Wnt signaling pathway genes in CRC in particular^35^. ChIP-seq experiments on Kbu on H4K5 and H4K8 in the context of sperm cell differentiation show histone butyrylation as a direct stimulator of transcription, while also competing with acetylation in chromatin reorganization^4,36^. H3K14bu ChIP-seq in mouse livers was recently shown to be associated with transcriptionally active chromatin and specifically with carboxylic acid and lipid metabolism^37^.

Compared to Kpr, fewer differentially bound regions were associated with H3K18bu and H4K12bu: 2305 and 793, respectively (Supplementary Fig. 8a, 9a). Interestingly, top scoring sites associated with both Kbu marks were lncRNAs *PRNCR1* and *PCAT1*, enhancers in the same chromosomal region as *MYC*, shown to play a pivotal oncogenic role in CRC^38,39^. *PRNCR1* showed a 3.4-fold (FDR = 2.25e-09) and 2.9-fold (FDR = 3.14e-77) increase in H3K18bu and H4K12bu binding, respectively (Supplementary Figs. 8b, f, 9b), whereas *PCAT1* showed a 4.19-fold increase in H3K18bu (FDR = 6.79e-16) and a 2.8-fold increase in H4K12bu binding (FDR = 1.57e-12). GO enrichment, differential motif analysis and integration with butyryl ATAC-seq results identified pathways similar to Kpr and propionyl ATAC-seq (Supplementary Figs. 8c-e, 9c-f, 10d-g).

**Fig. 3 |:**
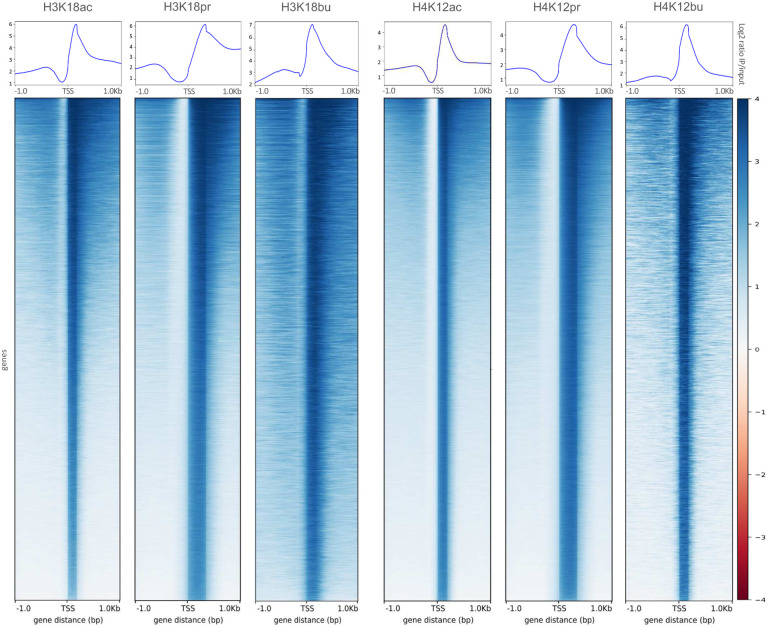
TSS distribution profiles of H3K18ac/pr/bu and H4K12ac/pr/bu associated ChIP-seq peaks as function of read coverage. *Upper panels*: Aggregate read density profile plots of genomic region distributions within +/− 1 Kb of TSS as a function of log2 IP/input ratio. *Lower panels*: Read density heatmaps of gene distributions with maximum (z = 4) and minimum (z = - 4) values of heatmap intensities. Plots generated by deepTools.

Next, we visualized the read coverage over genomic regions in 1 kB upstream and downstream of TSS for all our acyl lysine histone marks to evaluate global enrichment across all TSS (Fig. 3). In each case, we saw considerable enrichment of our marks proximal to the TSS and beyond, with increases in read density over input proportional to the length of the acyl lysine chain. Propionyl and butyryl marks showed significantly higher density distributions in the positive, downstream of TSS direction compared to their acetyl counterparts. All three types of acyl marks showed consistency in the distribution profiles (Fig. 3, upper panels) and heatmap densities (Fig. 3, bottom panels). These results indicate that Kpr and Kbu marks increased chromatin accessibility, compared to Kac. Feature distribution of differentially bound genes (+/− 3 kB of TSS) also showed increased chromatin accessibility and consistency in feature distributions among the three types of acyl marks, with a shift towards distal intergenic regions in Kpr and Kbu (Supplementary Fig. 11). These results indicate a greater role played by *cis-*regulatory elements and a more open chromatin structure.

Lastly, we examined functional differences between the three types of acyl marks by looking at annotated genes that were unique to each (Supplementary Fig. 12). Terms unique to Kac marks pointed to regulation of cell cycle and chromosome organization (Supplementary Fig. 12a, d). By contrast, terms unique to Kpr pointed to regulation of cation transport, organ morphogenesis and locomotion (Supplementary Fig. 12b, e). Elements unique to Kbu were associated almost exclusively with regulation of cell motility, migration and locomotion (Supplementary Fig. 12c). The results indicate that while there are some functional similarities between Kpr and Kbu marks, they are distinct from Kac. Looking at shared annotated features between Kpr/Kbu marks and ATAC/RNA-seq results we saw many of the same pathways, highlighting their functional similarities as well as their differences (Supplementary Fig. 13).

### SCFA increase chromatin accessibility in key regions of CRC cells

To address the effect of propionate and butyrate on chromatin accessibility at a more global level, we performed differential ATAC-seq. We hypothesized that NaPr and NaBu treatment would result in greater chromatin accessibility. Out of a total of 22,238 regions identified as differentially accessible, 18,404 (83%) sites showed positive fold-change in the NaPr-treated group compared to 3,834 in the untreated group (FDR < 0.05) (Supplementary Fig. 10a, top). Differentially accessible genes and GO pathways associated with NaPr treatment were consistent with Kpr ChIP-seq results, particularly with respect to genes controlling cell-substrate adhesion (*COL26A1*, 2.82-fold, FDR = 8.78e-37) as well as epithelial development (*KLF2*, 3.39-fold, FDR = 6.09e-37) and β-catenin-TCF complex assembly (Supplementary Fig. 10b, top). Sites that showed a reduction in accessibility were involved in cell cycle G1 arrest (*CDKN1A*, −1.78-fold, FDR = 9.04e-14), as well regulation of cell cycle and cell proliferation (*ZNF703,* −1.74-fold, FDR = 1.41e-11) (Supplementary Fig. 10a, top). Among differentially bound sites overall, the NaPr-treated group had a higher mean read concentration, indicating increased binding affinity under more open chromatin (Supplementary Fig. 10c, top).

By contrast, over three times as many sites were identified with butyryl ATAC-seq compared to propionyl ATAC-seq. Out of 71,318 sites identified as differentially accessible, 38,089 (53%) showed positive fold-change in the treated group and 33,229 underwent negative fold-change in the untreated group (Supplementary Fig. 10a, bottom). Genomic annotations identified genes involved in muscle contraction (*SSPN,* 2.31-fold, FDR = 5.41e-136), axon guidance and differentiation (*SEMA3D*, 2.38-fold, FDR = 5.16e-131), as well as actin and microtubule binding (*MYO3B*, 2.41-fold, FDR = 8.11e-99) (Supplementary Fig. 10b, bottom). GO pathway analysis showed enrichment in mesenchymal cell proliferation, metanephros development and fibroblast apoptotic process, as seen with Kbu ChIP-seq (Supplementary Fig 10b, bottom, Supplementary Fig. 8c, 9c).

Among sites that lost accessibility were genes encoding a zinc finger TF required for DNA damage-induced p53 activation (*CXXC5*, −2.06-fold, FDR = 1.26e-187) and a G protein subfamily member mediating transmembrane signaling (*GNAZ*, −3.04-fold, FDR = 4.76e-160). Among differentially accessible sites overall, the NaBu-treated group did not have a significantly higher mean read concentration, indicating increased accessibility in the treated group was offset by decreased accessibility in the untreated group (Supplementary Fig. 10c, bottom).

### SCFA affect gene expression in CRC cells

**Fig. 4 |:**
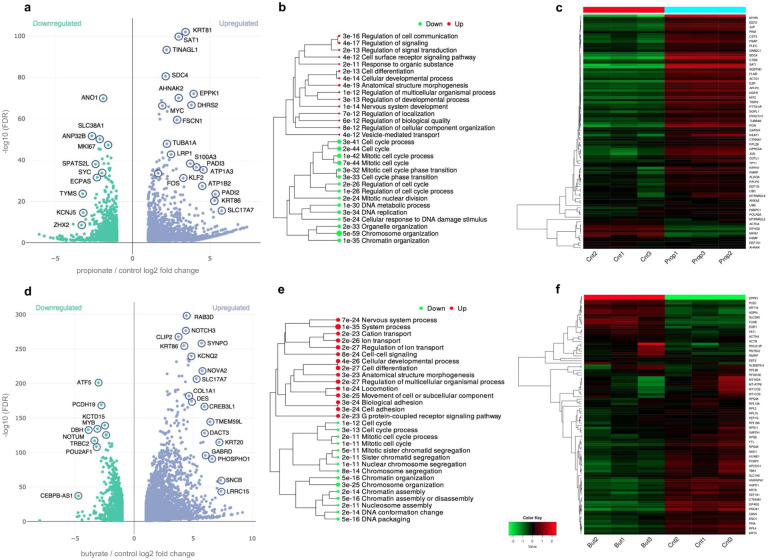
Propionyl and butyryl differential gene expression by RNA-seq. **a** Volcano plot showing gene upregulation vs downregulation in 10 mM NaPr treated vs control groups. **b** Hierarchical cluttering of GO ‘Biological Process’ terms of upregulated vs downregulated pathways in NaPr treated vs control groups. **c** Heatmaps of 50 most variable genes in NaPr treated vs control groups. **d** Volcano plot showing gene upregulation vs downregulation in 1 mM NaBu treated vs control groups. **e** Hierarchical cluttering of GO ‘Biological Process’ terms of upregulated vs downregulated pathways in NaBu treated vs control groups. **f** Heatmaps of 50 most variable genes in NaBu treated vs control groups. Differential expression analysis performed by DESeq2. Hierarchical clustering was performed using ShinyGO. Size of dots indicates statistically significant *P* values. n = 3 experimental replicates for each condition.

In order to get a more complete picture of SCFA induced alterations on the regulatory landscape of CRC, we examined global changes in gene expression by bulk RNA-seq. Differential gene expression analysis following NaPr treatment identified 2,027 upregulated and 1,151 downregulated genes (Fig. 4a). Among the genes showing significant upregulation, also identified as Kpr targets and showing increased accessibility by ATAC-seq were *SAT1* (2.99-fold FDR = 2.04e-100), *AHNAK2* (2.98-fold, FDR = 8.71e-71), *DHRS2* (3.80-fold, FDR = 2.44e-67), *KLF2* (3.28-fold, FDR = 4.95e-32) as well as *MYC* and *FOS* (Supplementary Fig. 4c, 5c). Among genes that underwent downregulation were mainly those involved in cell proliferation (*ANP32B*, −2.63-fold, FDR = 2.01e-52) and cell cycle progression (*MKI67*, −1.57-fold, FDR = 5.90e-48). Hierarchical clustering showed upregulation in receptor signaling, development, anatomical structure morphogenesis, and downregulation in cell cycle, cell division, and chromatin organization (Fig. 4b). The heatmap of top 50 most variable genes showed enrichment in *MYC, JUN* and *AHNAK2* (Fig. 4c).

Similarly, NaBu treatment led to upregulation of genes controlling ion transport, anatomical structure morphogenesis and cell adhesion, and downregulation in cell cycle and chromatin assembly (Fig. 4d–f). Taken together, our data point to a mechanism whereby the antiproliferative properties of propionate and butyrate in CRC can be attributed to their dysregulation of key CRC oncogenes such as *MYC*, *FOS* and *JUN*, as well their simultaneous triggering of downregulation of genes controlling cell cycle and cell division.

**Fig. 5 |:**
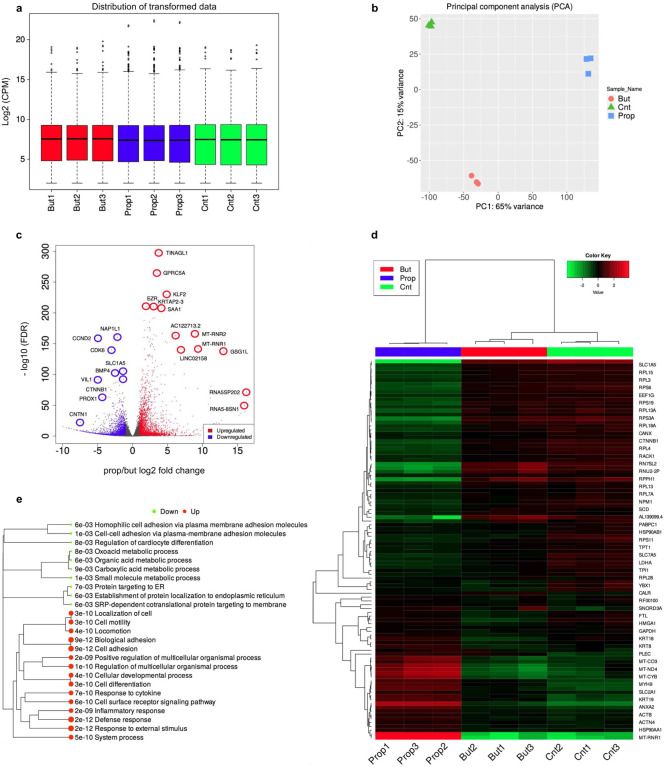
Propionyl/butyryl differential RNA-seq. **a** Distribution of log2 CPM (counts per million) transformed expression data for all conditions. **b** Principal component analysis of log2 CPM transformed expression data for all conditions. **c** Volcano plot of propionyl vs butyryl differential expression. **d** Heatmaps of 50 most variable genes for all three conditions. **e** Hierarchical cluttering of GO ‘Biological Process’ terms of differentially expressed pathways in propionyl vs butyryl RNA-seq. Pathways are clustered together based on shared genes. Size of dots indicates statistically significant *P* values. Hierarchical clustering was performed using ShinyGO. n = 3 experimental replicates for each condition. Normalization of raw counts performed by ‘cpm’ analysis in edgeR. Differential expression analysis performed by DESeq2.

To examine the differences in expression that were unique to propionate and butyrate we compared differential gene expression under both types of enrichments. Density distributions of counts were consistent across conditions and replicates within each condition (Fig. 5a). Principal component analysis (PCA) showed close clustering of replicates within each condition, with the most variation seen between different conditions forming separate clusters (Fig. 5b). Differential expression following NaPr versus NaBu treatment showed 3,082 upregulated and 2,783 downregulated genes (Fig 5c). The heatmap of top 50 most variable genes and hierarchical clustering of GO ‘Biological Process’ terms showed preferential enrichment of organic and carboxylic acid metabolism associated with NaBu treatment, while NaPr treatment showed enrichment in cell motility and locomotion, as well as cellular development and differentiation (Fig. 5d, e).

### Genomic localization of H3K18bu and H4K12bu in normal vs cancer cells shows enrichment in adherens junction assembly, transport and protein processing

We next examined the differences in genome-wide distribution of Kbu marks in CRC (SW480) versus normal (CCD841) cells following 1 mM NaBu treatment, particularly in relation to CRC-relevant genes. Out of 89,340 targets associated with H3K18bu differential binding in the two cell lines, 81,999 (92%) sites had higher binding affinity in normal cells, compared with 7,341 sites in cancer cells (FDR < 0.05) (Supplementary Fig. 14a). GO ‘Biological Process’ analysis in the normal cell line showed enrichment in cell-substrate and adherens junction assembly, as well as ion transport and protein processing (Supplementary Fig. 14b, top). By contrast, the same mark in the cancer cell line showed enrichment in mesenchymal cell proliferation, β-catenin-TCF complex assembly, and fibroblast apoptotic process (Supplementary Fig. 14b, bottom). Similarly, out of 64,886 targets associated with H4K12bu, 61,978 (96%) sites had higher binding affinity in normal cells, compared to 2,908 sites in cancer cells (Supplementary Fig. 14c). GO ‘Biological Process’ analysis in normal cells also showed enrichment in adherens junction assembly and transport but also regulation of chromatin silencing and H3K9 demethylation (Supplementary Fig. 14d, top). By contrast, GO analysis in the cancer cell line showed enrichment in mesenchymal cell proliferation, Wnt signaling pathway, and ER stress-induced apoptotic signaling (Supplementary Fig. 14d, bottom).

While both marks were associated with adherens and cell junction assembly, in SW480 cancer cells they were associated with mesenchymal cell proliferation, Wnt/β-catenin signaling, as well as apoptotic processes, while in normal cells they showed association with transmembrane ion and endosome to lysosome transport, as well as protein processing. Moreover, following NaBu treatment, many of the CRC-relevant genes monitored throughout the study, such as *MYC* and *FOSL1* showed a 3 to 7-fold reduction in Kbu binding affinity in the normal cell line compared to the CRC cell line (Supplementary Fig. 14e-h).

### Genomic localization of H3K18bu and H4K12bu in fiber-supplemented mouse intestines reveal targets involved in cell-substrate junction assembly and localization

**Fig. 6 |:**
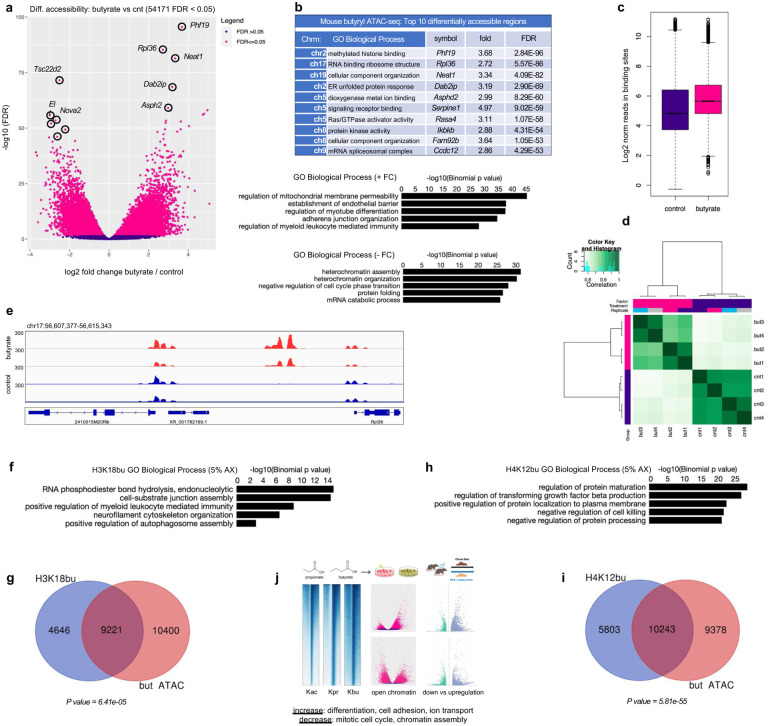
Mouse butyryl ATAC-seq and Kbu CUT&Tag in mouse intestines. **a** Differential accessibility at 1 mM NaBu treatment. Sites identified as significantly differentially accessible are shown in red. n = 4 experimental replicates for each condition. **b** Top ten differentially accessible regions associated with NaBu treatment sorted by false-discovery rate adjusted *P* value (FDR < 0.05), and top GO ‘Biological Process’ terms associated with positive vs negative fold change. **c** Normalized reads in binding sites at NaBu treatment. **d** Correlation heatmap showing clustering of replicates from NaBu treated vs untreated group. **f** Top GO ‘Biological Process’ terms associated with H3K18bu in HFS + 5% arabinoxylan group. **g** H3K18bu and butyryl ATAC-seq annotated peak overlap. **h** Top GO ‘Biological Process’ terms associated with H4K12bu in HFS + 5% arabinoxylan group. **i** H4K12bu and butyryl ATAC-seq annotated peak overlap. Significance of overlaps determined by hypergeometric test-generated *P v*alue. **j** Overview of SCFAs propionate and butyrate as regulatory elements affecting histone binding, chromatin accessibility and gene expression.

To further investigate the link between dietary fiber metabolism, chromatin accessibility and histone butyrylation, we performed ATAC-seq in CT26 mouse colorectal cells and cleavage under targets and tagmentation (CUT&Tag) on large intestine tissues from mice fed chow containing the dietary fiber arabinoxylan (AX, 5% w/w) (Supplementary Fig. 15). First, we examined differential chromatin accessibility following 1 mM NaBu treatment in CT26 cells. Out of 54,171 sites identified as differentially accessible, 39,956 (74%) gained accessibility following NaBu treatment, compared to 14,215 sites in the untreated group (FDR < 0.05) (Fig. 6a). Peak distributions, regulated genes and GO enrichments analysis showed similar results to those in SW480 and CCD841 cell lines (Fig. 6a–e).

Differential Kbu binding analysis of mouse intestines on a 5% arabinoxylan diet by CUT&Tag identified 21,665 sites associated with H3K18bu versus Kac, and 26,103 sites associated with H4K12bu versus Kac (FDR < 0.05) (Supplementary Fig. 16a, b, d, e). GO analysis and feature distributions were in agreement with Kbu ChIP-seq results in cells (Supplementary Figs. 16c, f, g, h, 11). Annotation of top H3K18bu-associated regions identified genes involved in cell proliferation and differentiation (*Fgf14*, 4.60-fold, *P* = 1.61e-05) as well as ion channel regulatory activity (*Akap9,* 5.72-fold, *P* = 3.55e-05) and actin filament polymerization (*Cyria,* 5.86-fold, *P* = 3.92e-05) (Supplementary Fig. 16b). GO analysis identified pathways controlling cell-substrate junction assembly, cytoskeletal organization as well as autophagosome assembly (Fig. 6f). Comparing genes from mouse butyryl ATAC-seq following NaBu treatment and H3K18bu targets *in vivo* revealed 9221 (72%) overlapping elements out of 13867 (*P* = 6.41e-05) (Fig. 6g). Similarly, top H4K12bu-associated regions were involved in fibroblast growth factor receptor activity (*Fgfrl1,* 6.97-fold, *P* = 5.33e-06) as well as tight junction cell adhesion activity (*Cldn3*, 5.72-fold, *P* = 5.33e-06) (Supplementary Fig. 16e). GO analysis identified pathways controlling protein processing, membrane localization, as well as TGF-β production (Fig. 6h). Comparing mouse butyryl ATAC-seq results following NaBu treatment to H4K12bu targets *in vivo* revealed 10,243 (64%) overlapping elements out of 16,046 (*P* = 5.81e-55) (Fig. 6i).

## DISCUSSION

The SCFAs propionate and butyrate are produced by the microbiome and have broad biological effects. To gain insights into how they may directly affect gene regulation and expression we have combined histone PTM profiling by tandem MS/MS with ChIP-seq, CUT&Tag, ATAC-seq and RNA-seq to understand the epigenetic regulatory function of these SCFA metabolites in CRC versus normal cells and *in vivo* (Fig. 6j). We identified the functional role of several propionyl- and butyryl-lysine modifications on H3 and H4 by identifying their genomic regions of interaction as well as their biological pathways. We have also shown the effect of these histone marks in promoting chromatin accessibility and changes in gene expression.

Our results link SCFA supplementation and increased chromatin accessibility by histone propionylation and butyrylation to changes in gene expression and regulation of anatomical structure morphogenesis, differentiation and ion transport. In the context of CRC, SCFA supplementation led to a homeostatic dysregulation via hyperactivation of Wnt/β-catenin as well as TGF-β signaling pathways and activation of *MYC, FOS* and *JUN* oncogenes. Our results point to a 3–4-fold decrease in binding affinity of both Kbu marks to these oncogenic targets in normal versus cancer cells. To our knowledge, this is the first instance supporting Kbu direct targeting of genomic regions controlling growth and differentiation, rather than inhibition of deacetylation. It is also, to our knowledge, the first time that lncRNAs like *PRNCR1*, *PCAT1* and *CRAT37* were reported to be associated with Kbu binding in colorectal cells. It is generally thought that in cancer cells under aerobic metabolism butyrate (and to some extent propionate) accumulates and acts as an HDAC inhibitor leading to apoptosis^40^. Here, we have expanded upon SCFAs’ role as unique regulatory elements and have shown the genome-wide localization of H3K18pr/bu and H4K12pr/bu in CRC cells, and in case of Kbu in mouse intestines as well. Our data point to a mechanism involving dysregulation of a broad range of oncogenes such as *MYC*, and tumor suppressing genes such as *TGF-βR2* and *SMAD2/3*. Our integrated multiomics data show the unique role of propionate and butyrate as regulators of histone acyl lysine levels leading to increased chromatin accessibility. In cancer cells, this results in overexpression of already high levels of proto-oncogenes controlling growth and differentiation, which may ultimately lead to cell death, especially in instances of elevated butyrate levels. This model extends the existing repertoire of SCFAs as epigenetic regulators beyond inhibitors of deacetylation. Regarding HDAC activity, especially in case of propionate, we have not seen significant increases in acetylation. In addition, for each Kpr and Kbu ChIP-seq experiment, we used the corresponding Kac marks as controls, to distinguish each histone mark’s unique targets under enrichment.

These data support a model that colonic SCFAs produced by microbial metabolism of fiber increase epithelial homeostatic gene expression pathways and impair carcinogenesis by direct histone modification. Given the rapid increase in colorectal cancers, especially in younger age populations recently, our results imply that dietary factors should be optimized to improve human health and diminish cancer onset. For example, the results raise the possibility of modulating histone post-translational modifications through dietary adjuvants, or through the creation of synthetic acyl chains to more precisely tune colonic epithelial gene expression. In addition, modulation of microbial populations or microbial metabolism may lead to improved epithelial homeostasis via epigenetic remodeling from microbial-derived acyl intermediates. Finally, given the high concentrations of SCFAs in the colonic environment, our results raise the possibility that chromatin-embedded acylations could serve as a storage depot of acylations that may be removed and further metabolized or recycled when colonic SCFA levels drop due to fiber limitation, or antibiotic usage that destroys microbial populations. Taken together, our results highlight the crucial importance of understanding mechanisms of SCFA utilization and point towards ways of utilizing SCFA epigenetic modifications to improve human health.

## METHODS

### Cell lines.

Adherent SW480, CCD841 and CT26 cells were obtained from ATCC (SW480 # CLL - 228, CCD841 CoN, # CRL - 1790, CT26.WT # CRL-2638) and cultured in commercially grown EMEM media containing 10 % fetal bovine serum (FBS)/1 % Pen/Strep (PS), or EMEM -glucose + 1 % PS (Sciencecell) and passaged every 3 – 5 days at 70 – 75 % confluence. CT26 cells were cultured in RPMI media containing 10 % FBS/1 % PS. Cells were treated with 0 – 10 mM NaPr and NaBu for 12 h. followed by cell counting and harvesting. All cell lines were maintained at 37° C (33° C in case of CCD841) in a humidified atmosphere containing 5 % CO_2_.

### Immunoblots.

Histones were first acid-extracted as described below. Protein extracts were made in RIPA buffer and quantitated by BCA assay and diluted to equal concentrations and mixed with 4x LDS sample buffer and sample reducing agent (Invitrogen). Polyacrylamide gel electrophoresis was performed on NuPAGE Novex gradient gels (Thermo Fisher) followed by wet transfer to nitrocellulose membranes. Blocking was briefly performed with 5 % non-fat milk and primary antibody was incubated overnight at 4° C in 5 % milk, followed by washing in PBST, then with HRP-conjugated secondary antibody (Cell Signaling) at room temperature for 1 hour followed by washing, then developed with ECL pico or femto kits (Thermo Fisher) and imaged using ChemiDoc Imaging system (Bio-Rad).

### Cell viability assays.

Cell viability in the presence of increasing levels of NaPr and NaBu was performed using the CellTiter-Blue^®^ Cell Viability Assay (Promega). TSA was used as a negative control. The assay measures the ability of living cells to convert a redox dye (resazurin) into a fluorescent end product (resorufin). Nonviable cells do not generate a fluorescent signal. 100 uL (or ~ 5,000 adherent cells) were plated in 96-well plates in EMEM media containing 10 % fetal bovine serum (FBS)/1 % Pen/Strep (PS). All cell lines were maintained at 37° C in a humidified atmosphere containing 5 % CO_2_. The media was aspirated after 24 h and replaced with media containing increasing levels of NaPr and NaBu (0 – 100 mM) and TSA (0 – 100 μM) in ten-fold increments with three replicates per condition. After 72 h, 40 uL of CellTiter-Blue^®^ reagent was added to each well and cells were incubated for 4 h. Following incubation, fluorescence at 560/590 nm was measured using Infinite M1000 Tecan i-control (v1.10.4.0) plate reader.

### Histone acid extraction.

SW480 and CCD841 colorectal cells were grown in EMEM media containing 10 % fetal bovine serum (FBS)/1 % Pen/Strep (PS). All cell lines were maintained at 37° C in a humidified atmosphere containing 5 % CO_2_. Prior to reaching confluence, cells were treated with increasing levels (0, 0.1, 1, 10 mM) of ^13^C3-Sodium Propionate (Cambridge Biosciences CLM-1865) for 12 h. Cells were harvested and pelleted at 1000 rpm for 5 min at 4° C. Cells were resuspended in TEB buffer (PBS, supplemented with: 0.5 % Triton X-100, 2 mM PMSF, 0.02 % NaN_3_) in 1 mL per 10^7^ cells. Cells were incubated on ice for 10 min with gentle stirring then centrifuged at 3,000 rpm for 5 min at 4° C. Pellets were resuspended in 200 uL of extraction buffer per 10^7^ cells. Cells were then incubated on ice for 30 min, centrifuged at 12,000 rpm for 5 min 4° C. The supernatant was acetone precipitated in 600 uL of acetone per 10^7^ cells and incubated overnight at – 20° C. Histones were then acid extracted and acetone precipitated. Pellets were air dried and saved for downstream MS analysis or resuspended in 100 uL of H_2_O and diluted to 1 μg or 10 μg levels for immunoblot analysis. Protein concentration was determined using Pierce^™^ BCA Protein Assay Kits (Thermo Scientific cat # 23225) with BSA as a standard.

### Mass spectrometric identification of histone propionylation.

Histone extracts were purified and derivatized according to Garcia et al.,^41^ and analyzed by nano-capillary liquid chromatography triple quadrupole mass spectrometry (nLC-QqQ MS). The digested and derivatized histone peptides were diluted in 0.1% TFA and injected to nLC-QqQ MS (Dionex nanoLC and a ThermoFisher Scientific TSQ Quantum). Peptides were first loaded to a trapping column (2 cm × 150 μm) and then separated with an analytical capillary column (10 cm × 75 μm). Both were packed with Magic C18 resin (Michrom). The chromatograph gradient was achieved by increasing percentage of buffer B from 2 – 35 % at a flow rate of 0.35 μL/min (A: 0.1 % formic acid in water, B: 0.1 % formic acid in acetonitrile) over 40 min. The peptides were then introduced into QqQ MS by electrospray from an emitter with 10 μm tip (New Objective) as they were eluted from capillary column. The QqQ settings were as follows: collision gas pressure of 1.5 mTorr; Q1 peak width of 0.7 (FWHM); cycle time of 3.5 s; skimmer offset of 10 V; electrospray voltage of 2.6 kV. Targeted histone PTM analysis was carried out using Multiple Reaction Monitoring (MRM), on a Thermo Scientific^™^ TSQ (Triple-Stage Quadrupole) instrument. Only specific precursor peptides were fragmented and specific product ion intensities were measured. Chromatographic separation and MS-measured intensities of different forms of peptides were used to distinguish modifications of interest. Exogenously introduced lysine propionylation was reported as ^13^C/^12^C heavy/light ratio (mean ± SD, n = 3). *p < 0.05, **p < 0.01, by ordinary, one-way ANOVA.

### ChIP-seq and data analysis.

For H3K18ac/pr/bu and H4K12ac/pr/bu ChIP-seq, cells were trypsinized and cross-linked with 1 % formaldehyde (EMD Millipore) for 10 min at RT. To quench the formaldehyde, 2 M glycine (Thermo Fisher Scientific) was added and incubated for 5 min at room temperature. Cells were washed with ice cold PBS twice, snap frozen and stored at − 80 °C. For ChIP-DNA preparation, cells were thawed by adding PBS and incubated at 4° C with rotation. Cells were treated with hypotonic buffer (20 mM HEPES pH 7.9, 10 mM KCl 1 mM EDTA pH 8.0, 10% glycerol) for 10 min on ice in the presence of protease inhibitors (G6521, Promega). Nuclear pellets were resuspended in RIPA buffer (Millipore) and incubated for 30 min on ice. Chromatin corresponding to 10 million cells for histone modifications was sheared with SFX250 Sonifier (Branson) 3 sets of 3 × 30 second sonications, set to intensity (output control) of 3.5. The lysates were transferred to Diagenode tubes, sonicate 16 rounds of 30 seconds on 30 seconds off, vortexing every 4^th^ round. Protein G beads (80 uL) and lysates were washed with RIPA buffer and immunoprecipitated with antibodies targeting H3K18ac (Abcam #ab40888), H3K18pr (PTM Bio #PTM-213), H3K18bu (Abcam #ab241458), H4K12ac (Abcam #ab46983), H4K12pr (PTM Bio #PTM-209), H4K12bu (# ab241120), 5 μg for each condition, at 4° C overnight on a nutator. For the input sample, 200 μl of sheared nuclear lysate was removed and stored overnight at 4° C. On the second day, supernatants containing ChIP-DNA and input was reverse crosslinked by incubating overnight at 65° C in 1% SDS, 1X TE buffer. On the third day, ChIP-DNA was treated with 250 μL 1X TE containing 100 μg RNase A (Qiagen) and 5.0 μL of 20 mg/mL Proteinase K (ThermoFisher Scientific) and then purified using Qiagen QIAquick purification columns. The ChIP-DNA samples were end-repaired using End-It DNA End Repair Kit (Lucigen) and A-tailed using Klenow Fragment and dATP (New England Biolabs). Illumina TruSeq adapters (Illumina) were ligated and libraries size-selected (200 − 400 bp) by gel extraction before PCR amplification. The purified libraries were subjected to paired-end sequencing on the Illumina HiSeq 4000/Novaseq 6000 SP to obtain an average of approximately 30 − 35 million uniquely mapped reads for each sample (Stanford Center for Genomics and Personalized Medicine, supported by NIH grants S10OD025212 and 1S10OD021763).

The resulting data were processed using the Kundaje Lab ChIP-seq ENCODE processing pipeline (https://github.com/kundajelab/chipseq_pipeline). Briefly, this pipeline takes FASTQ files for ChIP samples, and input as controls and outputs peak calls (bound regions; BR). Alignments to BR (GRCh38.p13) were performed using Bowtie2 (https://doi.org/10.1186/gb-2009-10-3-r25) and peak calling was performed using MACS2 (https://doi.org/10.1186/gb-2008-9-9-r137). Peak flies were analyzed for differential binding using DiffBind R package v.2.4.8 to produce a count matrix with an FDR-adjusted *P* value cutoff of < 0.05. Data visualization was performed in R as well as using the Integrative Genomics Viewer (http://www.broadinstitute.org/igv/). Differential motif enrichment analysis in HOMER (http://homer.ucsd.edu/homer/) was performed using function findMotifsGenome with default parameters to search for motif enrichment in the full accessible regions, and control IP and/or input used as background. Annotation was performed using ChIPpeakAnno and ChIPseeker R packages. TSS distribution heatmaps and profiles were determined using computeMatrix, plotHeatmap and plotProfile functions within deepTools v.3.1.0 (https://deeptools.readthedocs.io/en/develop/index.html). Genomic coordinate overlaps were determined using the intersect ‘function’ in bedtools, whereby the original entry in one set was reported once if any overlaps were found in the second set, effectively reporting that at least one overlap was found (https://bedtools.readthedocs.io/en/latest/content/tools/intersect.html). Gene Ontology analysis was performed using GREAT (http://great.stanford.edu/public/html/index.php) and ShinyGO (DOI: 10.1093/bioinformatics/btz931). Images in KEGG pathway analysis were produced by PATHVIEW(https://bioconductor.org/packages/release/bioc/html/pathview.html).

### ATAC-seq and data analysis.

ATAC-seq was performed using Omni-ATAC as described in Buenrostro et al.,^42^. Briefly, 50,000 viable cells were pelleted at 500 RCF for 5 min at 4° C. Cells were resuspended in 50 μl of ATAC-Resuspension Buffer (RSB) containing 0.1 % NP40, 0.1 % Tween-20, and 0.01 % Digitonin and mixed by pipetting. Following incubation on ice for 3 min, cells were washed with 1 mL cold RSB containing 0.1 % Tween-20 but no NP40 or Digitonin. Nuclei were pelleted at 500 RCF for 10 min at 4° C. Cells were resuspended in 50 μL of transposition mix containing 25 μL 2X TD buffer, 2.5 μL transposase (Illumina Tagmented DNA enzyme and buffer kit, Ref 20034210) (100 nM final), 16.5 μL PBS, 0.5 μL of 1 % Digitonin, 0.5 μL 1 % Digitonin, 0.5 μL of 10 % Tween-20, and 5 μL H_2_O). The final reaction was incubated at 37° C for 30 min in a thermomixer with 1000 RPM mixing. Pre-amplified transposed fragments were cleaned using the Zymo DNA Clean and Concentrator-5 Kit (cat # D4013). DNA was eluted in 21 μL of elution buffer. Samples then underwent 5 cycles of PCR using NEBNext 2x MasterMix. Each reaction contained 2.5 μL of 25 μM i5 primer, 2.5 μL of 25 μM i7 primer, 25 uL of 2x NEBNext master mix, and 20 μL of transposed/cleaned-up sample. Reactions were PCR amplified (72° C for 5 min, 98° C for 30 sec, followed by 5 cycles of (98° C for 10 min, 63° C for 30 sec, 72° C for 1 min) then held at 4° C. Using 5 μL of pre-amplified mixture, 15 μL qPCR (Applied Biosystems, Quantstudio 6 Flex) was then performed to determine the additional number of cycles. The conditions for qPCR were: 3.76 μL sterile H_2_O, 0.5 μL 25 μM i5 primer, 25 μM i7 primer, 0.24 μL 25x SYBR gold (in DMSO), 5 μL 2x NEBNext master mix and 5 μL of pre-amplified sample. Cycling conditions for qPCR were 98° C for 30 sec, followed by 20 cycles of (98° C for 10 sec, 63° C for 30 sec, 72° C for 1 min). qPCR amplification profiles were then manually assessed to determine the required number of additional cycles. Using the remainder of the pre-amplified DNA, 2 – 3 additional cycles were performed. Final PCR reactions were purified using Zymo DNA Clean and Concentrator-5 Kit (cat # D4013) and eluted in 20 μL of H_2_O. Amplified DNA library concentration was determined using Qubit 4 Fluorometer (Invitrogen). Library quality and size was assessed on an Agilent Bioanalyzer 2100 system using a high-sensitivity DNA kit. Multiplexed libraries were paired-end sequenced on the Illumina HiSeq 4000/Novaseq 6000 SP to obtain an average of approximately 50 million uniquely mapped reads per sample (Stanford Center for Genomics and Personalized Medicine, supported by NIH grants S10OD025212 and 1S10OD021763). The resulting data were processed using the Kundaje Lab ENCODE ATAC-seq processing pipeline (https://github.com/kundajelab/atac_dnase_pipelines). Briefly, this pipeline takes FASTQ files as input and outputs peak calls (accessible regions, AR). Alignments to AR (GRCh38.p13) were performed using Bowtie2 (https://doi.org/10.1186/gb-2009-10-3-r25) and peak calling was performed using MACS2 (https://doi.org/10.1186/gb-2008-9-9-r137). Peak flies were analyzed for differential accessibility using DiffBind v.2.4.8 R package to produce a counts matrix with an FDR-adjusted *P* value cutoff of < 0.05. Venn diagrams generated by https://bioinformatics.psb.ugent.be/webtools/Venn/.

### CUT&Tag and data analysis.

CUT&Tag analysis of NaBu treated CCD841 cells and mouse intestinal samples were performed using CUT&Tag-IT^™^ assay kits (Active Motif # 53160, 53170). Approximately 30 mg of tissue was homogenized per reaction by first chopping frozen tissue into smaller pieces in a petri dish with a razor blade on dry ice. 1 mL of CUT&Tag-IT Lysis Buffer (50 mM Tris pH 8.0, 10 mM EDTA, 0.4 % w/v SDS, 0.1 % protease inhibitor cocktail) was added per 10 mg of tissue and samples were further cut and minced in bulk. Samples were then Dounced 20 times with a loose pestle and 10 times with a tight pestle. Samples were then filtered with a 40 μM strainer in 15 mL conical tubes. Samples were centrifuged at 500 × g for 5 min at 4 °C. Samples of nuclei suspension were counted and normalized to ~ 500,000 nuclei per reaction. CCD841 cells (~ 500,000 / reaction) treated with NaBu for 12 h were harvested by centrifugation at 600 × g at RT. Both CCD841 cells and mouse tissue extracts were washed with 1X Wash Buffer (1 M HEPES pH 7.5, 1.5 mL 5 M NaCl, 12.5 μL 2 M spermidine), containing protease inhibitor cocktail (10 μL per 1 mL of Wash Buffer), and resuspended in Concanavalin A Beads slurry in a 1X Binding Buffer (1 M HEPES pH 7.5, 100 μL 1 M KCl, 10 μL 1 M CaCl_2_ and 10 μL 1M MnCl_2_). Cells and beads were incubated on an end-over-end rotator for 10 min. Cells were resuspended in ice-cold Antibody Buffer containing 2 mL Dig-Wash buffer (5 % digitonin with 40 mL 1X Wash buffer with PIC) mixed with 8 μL 0.5 M EDTA and 6.7 μL 30 % BSA not to exceed 500,000 cells per 50 μL reaction volume. 5 μg of undiluted primary antibody was added to each sample for an overnight incubation at 4° C with orbital mixing. 100 μL of Guinea Pig Anti-Rabbit secondary antibody diluted 1:100 in Dig-Wash buffer was then added to each reaction and the samples were incubated on an orbital rotator for 60 min at RT. 100 μL of 1:100 diluted CUT&Tag-IT^™^ Assembled pA-Tn5 Transposomes in Dig-300 Buffer (1 M HEPES pH 7.5, 3 mL 5 M NaCl and 12.5 μL 2 M spermidine with 5 % digitonin and 0.01 % protease inhibitor cocktail) was added to each sample and reactions were incubated on an orbital rotator for 60 min at RT. Following three washes with Dig-300 buffer, 125 μL of Tagmentation Buffer (5 mL Dig-300 buffer and 50 μL 1 M MgCl2) was added to each sample and reactions were incubated at 37° C for 1 h. To stop the tagmentation and solubilize DNA fragments, each sample received 4.2 μL of 0.5 M EDTA, 1.25 μL of 10 % SDS, 1.1 μL of Proteinase K (10 mg/mL). Samples were mixed and incubated at 55° C for 60 min. Following washing, samples were centrifuged at 17,000 × g for 2 min. PCR amplification was performed by adding 30 μL of Tagmented DNA from each reaction to 1 uL of dNTPs (10 mM), 0.5 μL of NEBNext Q5 High-Fidelity DNA Polymerase, 10 μL 5X Q5 Reaction Buffer, 3.5 μL of nuclease-free H2O and 2.5 μL of unique combinations of Nextera i5 and i7 indexing primers to a total of 50 μL reaction. PCR was performed using the following program on a thermal cycler with a heated lid: Cycle 1: 72° C for 5 min (gap filling) Cycle 2: 98° C for 30 sec Cycle 3: 98° C for 10 sec Cycle 4: 63° C for 10 sec. Repeated Cycles 3 – 4 14 times. Held at 72° C for 1 min and held at 10° C. PCR reactions were purified using Zymo DNA Clean and Concentrator-5 Kit (cat # D4013) and eluted in 20 μL of nuclease-free H_2_O. Amplified DNA library concentration was determined using Qubit 4 Fluorometer (Invitrogen). Library quality and size was assessed on an Agilent Bioanalyzer 2100 system using a high-sensitivity DNA kit. Multiplexed libraries were paired-end sequenced on the Illumina HiSeq 4000 / Novaseq 6000 SP to obtain an average of approximately 50 million uniquely mapped reads per sample (Stanford Center for Genomics and Personalized Medicine, supported by NIH grants S10OD025212 and 1S10OD021763). The resulting data were processed using the Kundaje Lab ENCODE ATAC-seq processing pipeline (https://github.com/kundajelab/atac_dnase_pipelines). Briefly, this pipeline takes FASTQ files as input and outputs peak calls (accessible regions, AR). Alignments to AR (GRCh38.p13) were performed using Bowtie2 (https://doi.org/10.1186/gb-2009-10-3-r25) and peak calling was performed using MACS2 (https://doi.org/10.1186/gb-2008-9-9-r137). Peak flies were analyzed for differential accessibility using DiffBind v.2.4.8 R package to produce a counts matrix with an FDR-adjusted *P* value cutoff of < 0.05.

### RNA-seq and data analysis.

RNA was extracted using the RNeasy Mini Kit (#74136, Qiagen). Libraries were prepared using NEBNext Ultra II RNA library prep kit for Illumina (# E7770) and rRNA depletion kit (# E6310). Paired-end sequencing on the Illumina Novaseq 6000 SP yielded an average of approximately 20 million uniquely mapped reads per sample for mRNA-seq (Stanford Center for Genomics and Personalized Medicine, supported by NIH grants S10OD025212 and 1S10OD021763). The resulting data were aligned to the human genome (GRCh38.p13) by STAR v.2.5.4b (https://github.com/alexdobin/STAR). The aligned transcripts were quantitated based on features in the GENCODE annotation database (GRCh38.p13) by RSEM v.1.3.1 (http://deweylab.biostat.wisc.edu/rsem/). Differentially expressed genes were detected using DESeq2 v.1.20.0 R package with a FDR-adjusted *P* value cutoff of < 0.05 (https://bioconductor.org/packages/release/bioc/html/DESeq2.html). Venn diagrams generated by https://bioinformatics.psb.ugent.be/webtools/Venn/.

### Animal handling and preparation of intestinal tissue samples.

All experiments were approved by and conducted in strict accordance with Stanford University’s Administrative Panel on Laboratory Animal Care (APLAC 34017). Male CETP-ApoB-100 transgenic mice were procured from Taconic Biosciences (New York, USA). All animals were housed in a temperature-controlled, specific pathogen-free environment, with a 12:12 light-dark cycle, temperature 24 ± 1° C and humidity ranging between 40 – 60 %. Food and water were available *ad libitum*. Mice were assigned randomly to two dietary groups: 1) low fat control diet (10% fat, TD. 08485, Envigo Teklad, Madison, WI, USA), or a 2) high fat, high sucrose diet (42% fat, TD 88137, Envigo Teklad, Madison, WI, USA). The high fat, high sucrose (HFS) diet was selected to mimic the *western diet* aiming to characterize and augment atherosclerosis development in the ApoE-deficient transgenic mouse. Over a span of 4 weeks, both groups were subjected to their respective diets. Following the initial 4-week duration, the control group continued on their diet for an additional 4 weeks. In contrast, the HFS group received a supplementation of 5 % arabinoxylan (5 % w/w of total HFS, blended in-house with their powdered HFS diet) for an additional 4 weeks. At the conclusion of this second 4-week period, all mice underwent a 6-hour fast and were then anesthetized with isoflurane and subsequently sacrificed via cervical dislocation. The large intestine was dissected, weighed, flash frozen in liquid nitrogen and subsequently stored in – 80° C until analysis.

### Statistics.

Details of statistical tests are provided in the figure legends and manuscript text, including the tests used, definition and quantification of n, and quantification of measurement precision. Multiple-hypothesis testing was used for high-throughput sequencing studies and pathway analysis with and FDR cutoff of at least < 0.1 required to declare statistical significance. Otherwise, statistical significance was declared for *P* values < 0.05.

## Data Availability

ChIP-seq, ATAC-seq and CUT&Tag raw data and differential peak call files have been uploaded to GEO with accession numbers GSE252649, GSE252652 and GSE252754, respectively. RNA-seq data are deposited in GEO with accession number GSE252753. GSE252653 is the reference for the Series of four data sets.
